# Stereotactic Gamma Knife® Radiosurgery of Intraocular Retinoblastoma: Six-Year Experience

**DOI:** 10.7759/cureus.28751

**Published:** 2022-09-03

**Authors:** Andrey A Yarovoy, Andrey V Golanov, Vera A Yarovaya, Valery V Kostjuchenko, Denis P Volodin

**Affiliations:** 1 Ocular Oncology and Radiology, S. Fyodorov Eye Microsurgery Federal State Institution, Moscow, RUS; 2 Department of Radiation Oncology, N.N. Burdenko National Medical Research Center for Neurosurgery, Moscow, RUS; 3 Gamma Knife, Neurosurgery Business Center, Moscow, RUS

**Keywords:** radiotherapy, eye salvage treatmemt, radiosurgery, gamma-knife, retinoblastoma

## Abstract

Background

External beam radiotherapy for resistant retinoblastoma is now seen as a last resort to saving the eye because of the risk of severe side effects: secondary cancers and cosmetic problems of orbital bone growth retardation. To reduce such complications, treatment modalities have shifted towards new radiation therapy techniques. No information on single fraction Gamma Knife® radiosurgery (GKRS) for intraocular retinoblastoma exists.

Materials and methods

Eighteen children (19 eyes) with retinoblastoma were treated with GKRS. The mean age at the time of treatment was 35 months (from 12 to 114 months). Before GKRS, all routes of chemotherapy delivery were held in all cases. The eligibility criteria for GKRS were retinoblastomas not amenable either to systemic or local chemotherapy and local ophthalmological treatment, retinoblastomas too large for conventional local methods, and inability to perform intraarterial chemotherapy. Conventional external beam radiotherapy was excluded in the presented cases, given the possible complications mentioned above. In every case, eye removal was suggested to the child's parents, but they flatly refused. GKRS was proposed as the last chance to save the eye (in four cases, it was performed on the only eye). The median prescribed dose was 22 Gy (interquartile range [IQR]: 18-35 Gy), and the median prescribed isodose was 50% (IQR: 36-90%).

Results

Local control was achieved in 79% of cases (complete tumor regression in 69%, incomplete regression in 10%). Two eyes (10.5%) could not be preserved and had to be enucleated due to the tumor recurrence. Two eyes (10.5%) developed secondary complications (total vitreous hemorrhage, retinal detachment, and iris neovascularization), making adequate tumor control nearly impossible. Overall, 15 eyes (79%) were preserved, and four eyes (21%) were enucleated after GKRS with no signs of tumor recurrence and metastasis in the mean follow-up of 41 months. No acute radiation side effects occurred in any patient after GKRS. Ten children (10 eyes, 53%) were diagnosed with vitreous hemorrhage from mild to severe. Three eyes presented with optic neuropathy one year after GKRS, and four eyes developed retinopathy. Radiation-induced cataract occurred in two eyes. There were no cases of secondary glaucoma or keratopathy in our study. All patients and eyes treated by GKRS were stable within 41 months (from seven to 74 months).

Conclusions

Single fraction Gamma Knife® radiosurgery may be a reasonable salvage treatment for resistant and recurrent retinoblastoma as an alternative approach to enucleation in selected cases. GKRS should be considered in retinoblastoma management.

## Introduction

Retinoblastoma (Rb) is the most common devastating, blinding, and life-threatening intraocular tumor among children, usually presenting before five years of age [1]. The main focus of Rb management is the protection of life, but globe salvage and sight-saving are also considered. 

Management of Rb has changed considerably over recent years. As a first-line approach for children with Rb, chemotherapy is used and can be delivered by intravenous, intra-arterial, intravitreal, and sub-tenon routes. Local treatment options, such as laser photocoagulation, thermotherapy, cryotherapy, and brachytherapy, help in the management of eyes with Rb previously unsuccessfully treated with chemotherapy [2]. However, in some cases, Rb remains resistant to chemotherapy and all available local methods. Based on this, the new techniques of radiation therapy seem to be very promising.

Conventional external beam radiotherapy (EBRT), which has typically been used for resistant Rb treatment [3-6], is now seen as a last resort to save the eye because of the risk of late side effects - especially secondary radio-induced cancers, as well as cosmetic problems of orbital bone growth retardation due to a significant amount of healthy periocular tissues exposed to high radiation doses [7-11]. To reduce such complications, treatment modalities have shifted towards new radiation therapy techniques with the possibility of creating a small high-precision radiation field sparing surrounding healthy tissues. These modalities include intensity-modulated radiotherapy, stereotactic conformal radiotherapy, and proton beam radiation therapy [12-19]. To the best of our knowledge, no information on single fraction Gamma Knife® radiosurgery (GKRS) for intraocular Rb exists. Here, we present the six-year experience of GKRS in children with Rb.

## Materials and methods

In this retrospective study, we identified all patients treated with GKRS at our institution from February 2015 to February 2021 with at least seven months of follow-up. All included patients were pretreated Rb eyes, with GKRS being a rescue therapy for failure or lack of feasibility of all other established treatment modalities with parents' rejection of enucleation. The study was approved by S. Fyodorov Eye Microsurgery Federal State Institution Local Ethic Committee (approval #76.3). 

Before GKRS, all routes of chemotherapy delivery were held in all cases: systemic intravenous chemotherapy (IVC) in 16 patients (from two to six courses), intraarterial chemotherapy (IAC), and intravitreal chemotherapy (IVtC) in 18 eyes of 17 patients (from one to seven courses of IAC and from one to 12 courses of IVtC). Eight eyes of seven patients prior to GKRS underwent local treatment modalities such as transpupillary thermotherapy in seven cases, cryotherapy, and beta-ray brachytherapy in two cases, respectively. Six eyes presented vitreous seeds that did not respond to intravitreal chemotherapy (IVCT), 10 eyes had tumors confined to the retina, and three eyes were affected both by retinal and vitreous tumors. One patient was ineffectively treated with stereotactic Cyber-Knife® radiosurgery before GKRS.

The eligibility criteria for GKRS were retinoblastomas not amenable either to systemic or local treatment, retinoblastomas too large for conventional local methods, inability to perform intraarterial chemotherapy due to systemic and periocular side effects after the previous course in two cases (such as stroke and ophthalmic artery spasm) or systemic hemostasis disorders (Hagemann disease) in one patient.

We analyzed 19 eyes of 18 patients with Rb, eight were male, and 10 were female. The mean age at the time of GKRS was 35 months (from 12 to 114 months). Seven children had familial retinoblastoma with a proven RB1 mutation (44%). At initial presentation, patients were classified using the International Classification System for Retinoblastoma (ICRB) as follows: four eyes presented with ICRB group B (21%), one eye with ICRB group C (6%), 14 eyes with ICRB group D (73%). In four cases, GKRS was performed on the only eye. The mean follow-up time was 41 months (from seven to 74 months). The patient characteristics are listed in Table 1.

**Table 1:** General data of patients treated with GKRS and treatment outcomes ICRB - International Classification of Retinoblastoma; IAC - intra-arterial chemotherapy; IVitC - intravitreal chemotherapy; EPB - episcleral plaque brachytherapy; RD - retinal detachment; VH - vitreous hemorrhage; ON - optic neuropathy; RSC - retrobulbar space catheterization; PPV with MI - pars plana vitrectomy with melphalan irrigation.

Patient/eye	Age at treatment, months	Unilateral Rb - 1/ bilateral Rb - 2	The only eye	ICRB group	Previous treatment	Indications for GKRS	Planning target volume location	Complications after GKRS	Additional treatment	Outcome (eye salvage)	The follow-up period after GKRS, months		
1	41	2	yes	D	Chemotherapy, IAC, IVitC, thermotherapy	Resistance	Retina, vitreous lesions	VH	RSC	yes	41		
2	32	1		C	Chemotherapy, IAC, IVitC	Resistance	Retina	ON, retinopathy, cataract	RSC	yes	46		
3	21	2		B	Chemotherapy, IVitC, thermotherapy, cryotherapy	Hageman's disease	Retina	ON, retinopathy	RSC	yes	42		
4	21	2		B	Chemotherapy, thermotherapy	Hageman's disease	Retina	VH, RD	RSC, PPV with MI	yes	42		
5	13	2		D	IAC, IVitC, thermotherapy	Stroke after IAC	Retina	VH, cataract	RSC, PPV with MI	yes	50		
6	12	2	yes	D	Chemotherapy, IAC	Resistance	Retina	VH, RD	RSC	no	7		
7	114	1		D	Chemotherapy, IAC, IVitC	Resistance	Retina, vitreous lesions			yes	60		
8	43	1		D	Chemotherapy, IAC, IVitC	Tumor progression	Vitreous lesions	VH	RSC, PPV with MI	yes	74		
9	59	1		D	Chemotherapy, IVitC, thermotherapy	Resistance	Retina, vitreous lesions	RD		yes	46		
10	31	1		D	Chemotherapy, IAC, IVitC	Resistance	Vitreous lesions	VH, cataract		yes	61		
11	35	1		D	Chemotherapy, IAC, IVitC, thermotherapy	Tumor progression	Retina	Recurrence of RB		no	50		
12	26	2		B	Chemotherapy, IAC, IVitC, thermotherapy, cryotherapy, EPB	Ophthalmic artery spasm	Retina	VH, ON		yes	62		
13	34	2	yes	B	Chemotherapy, IAC, IVitC, EPB	Resistance, tumor recurrence	Vitreous lesions			yes	35		
14	30	1		D	IAC	Resistance, tumor recurrence	Vitreous lesions	Recurrence of RB		no	35		
15	14	2		D	Chemotherapy, IAC, IVitC	Resistance	Retina	VH, RD	RSC	no	40		
16	28	2		D	IAC, IVitC	Resistance	Vitreous lesions			yes	35		
17	36	2		D	Chemotherapy, IAC	Resistance	Retina			yes	31		
18	10	2	yes	D	Chemotherapy, IAC, IVitC	Resistance	Vitreous lesions	VH, retinopathy	RSC, PPV with MI	yes	17		
19	33	1		D	Chemotherapy, IAC, IVitC	Resistance	Retina	VH, retinopathy	RSC	yes	12		

Before GKRS was planned, all patients underwent an extensive ophthalmological examination under general anesthesia in order to record and document the exact extent of the tumors intended to treat. Treatment was performed under general anesthesia as well with Leksell Gamma Knife® (LGK) Perfexion® accompanied with Leksell Gamma Plan® (LGP) v.10 software for treatment planning, and later with LGK Icon™ accompanied with LGP v.11 and onboard stereotactic roentgen cone-beam computer tomographic scanner (CBCT). On the day of treatment, the eye was immobilized by fixing four rectus muscles with sutures, and the Leksell® stereotactic frame was fixed under local anesthesia with Naropin®. Then magnetic resonance imaging (MRI) with the 1 mm slice thickness was used for target and organs at risk (OAR) delineation for treatment planning. Our current MRI protocol included T1 with fat saturation (FatSat) without and with gadolinium enhancement, T1 without FatSat after gadolinium, and T2 fast spin echo (FSE) sequences. In most cases, roentgen computed tomography (CT) was also performed. In LGP v.10 1.25-mm slice thickness, CT data was used for accurate skull and external eye boundaries definition. In LGP v.11, MRI was used for this purpose, and we fulfilled CBCT before treatment for checking geometries using the onboard system. CT and later CBCT studies were also used to delineate orbital bones for future risk analysis.

In cases of vitreous seeding, the planned target volume (PTV) included either its entire volume or a quadrant of its exclusive lesion. In order to compensate occasional eye movement, the borders of retinal tumors were delineated with the near 1 mm capture of healthy tissues based on MRI, CT, and pediatric retinal camera (RetCam III Clarity Medical Systems, Pleasanton, USA) images. In one patient, 3 mm of the retrobulbar part of the optic nerve was included in PTV due to the lack of confidence in its involvement. When an eye was affected by both tumors confined to the retina and vitreous seeds, two PTVs were determined separately for vitreous and retinal lesions. Target volumes and OAR (e.g., lens, ciliary body, macula area, orbital bones, and optic nerve) were delineated by ophthalmologists, neuroradiosurgeon, and a medical physicist. Whenever possible, doses to OAR were reduced as much as possible. The mean procedure duration was 81 minutes (from 11 to 141 minutes).

In most cases, a prescribed dose for PTV margin was 22-24 Gy, which made up 50% of the maximum dose. A dose of 24 Gy was used only at the first stage of the study. The median prescribed dose was 22 Gy (from 18 to 35 Gy), and the median prescribed isodose was 50% (from 36 to 90%). The prescribed dose of 35 Gy was used in one patient due to the very high isodose (90%) and small PTV. PTV varied from 0.056 to 4.305 cm^3^. Maximum doses for OAR were the following: optic nerve head - from 8.2 to 38.4, the macula - from 11.3 to 40.3, ciliary body - from 1.9 to 24.4, lens - from 2.1 to 18.3, orbital bones - from 0.0 to 5.4. The patients were observed every two months for the first six months, then every three months, and after complete tumor control was achieved, at least once every six months.

## Results

In all cases, frame fixation, eye immobilization, and the procedure of GKRS itself were well-tolerated in the intra- and post-treatment periods. Complete tumor regression of both retinal and vitreous lesions was achieved in 11 eyes (69%) within one to 12 months (mean of six months). Incomplete regression was observed in two eyes (10%). Two patients (10%) presented with tumor recurrence, one of them underwent enucleation three months after GKRS, while the second one was inefficiently treated by repeated GKRS with subsequent enucleation nine months after GKRS. Within the histopathological specimens, viable tumor of standard risk was found in both eyes, and they did not require either adjuvant chemotherapy or EBRT. Two eyes (10%) developed secondary complications (total vitreous hemorrhage, retinal detachment, and iris neovascularization), making adequate observation of the tumor nearly impossible. These eyes were enucleated in the mean 10-month interval after GKRS. Histologic examination showed no viable tumor cells in both cases.

Examples of complete tumor regression after GKRS and treatment planning are shown in Figures 1-5 in the patient with a massive vitreous lesion and in Figures 6-9 in the patient with a large retinal tumor.

**Figure 1:**
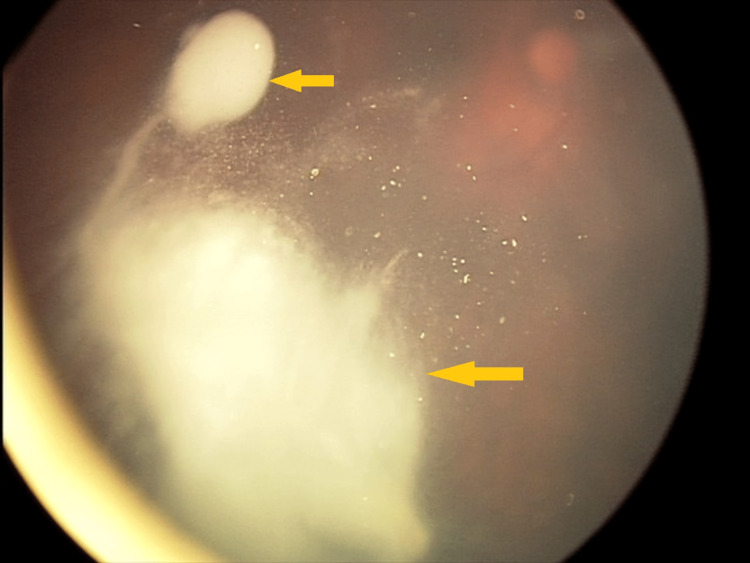
Massive vitreous lesion before GKRS GKRS - Gamma Knife® radiosurgery

**Figure 2:**
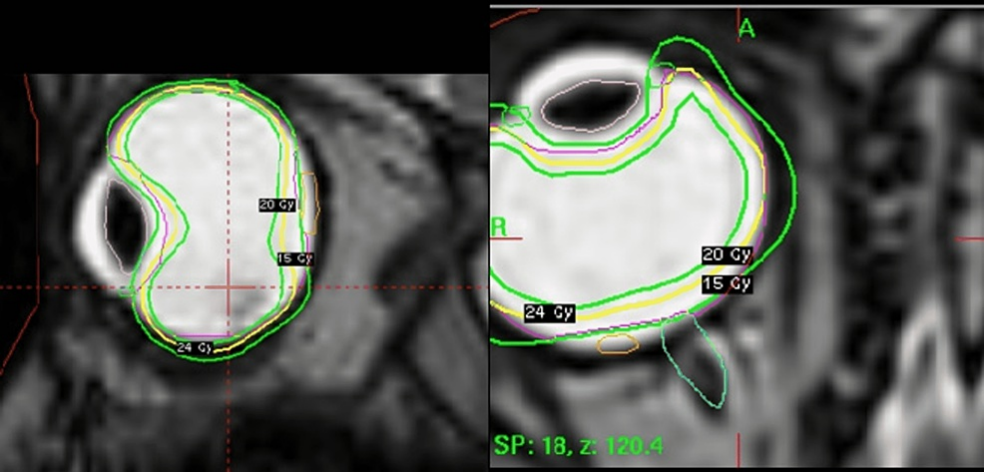
Treatment planning

**Figure 3:**
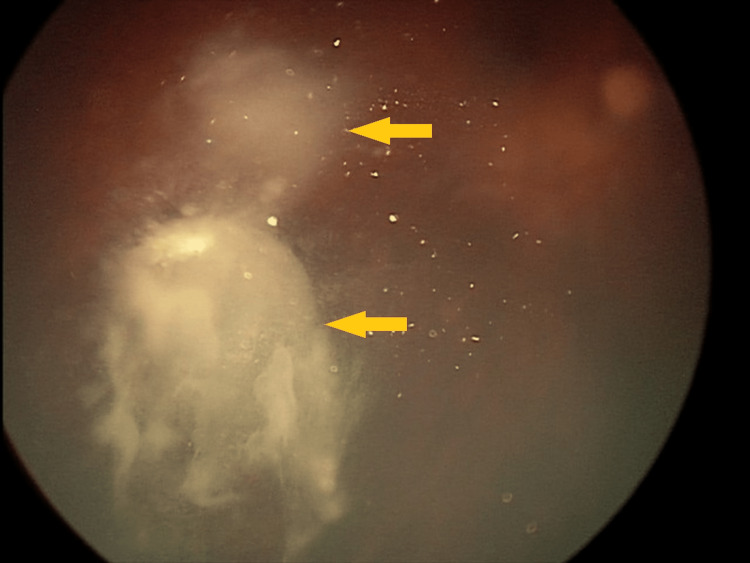
Incomplete tumor regression three months after GKRS GKRS - Gamma Knife® radiosurgery

**Figure 4:**
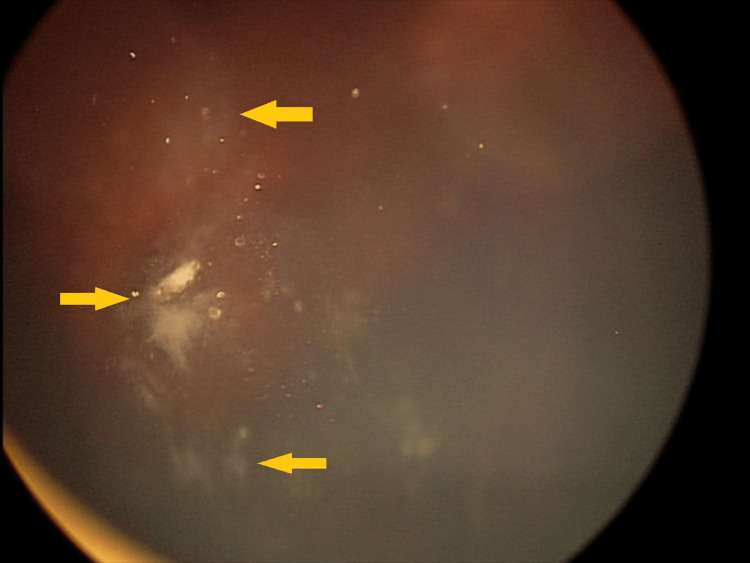
Incomplete tumor regression eight months after GKRS GKRS - Gamma Knife® radiosurgery

**Figure 5:**
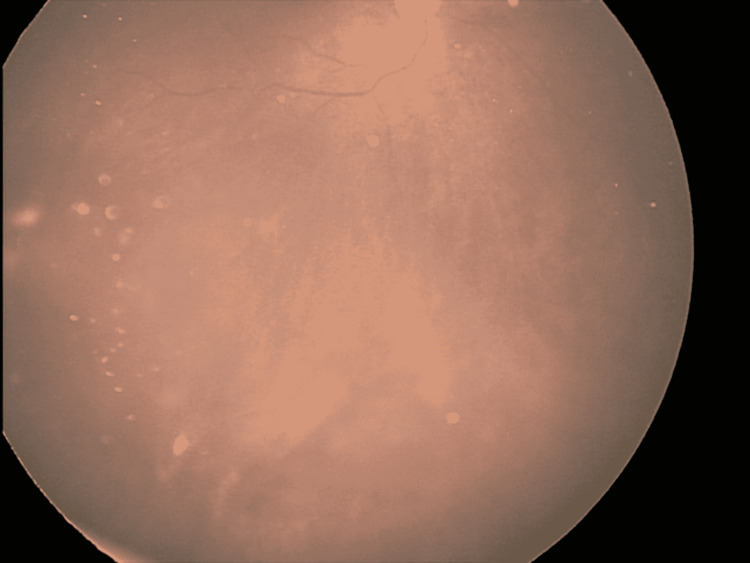
Complete tumor regression 10 months after GKRS GKRS - Gamma Knife® radiosurgery

**Figure 6:**
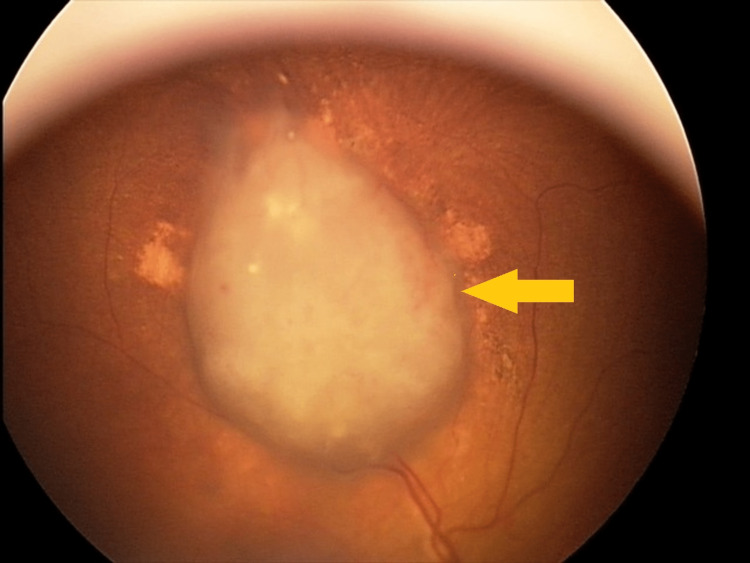
Large retinal tumor before GKRS GKRS - Gamma Knife® radiosurgery

**Figure 7:**
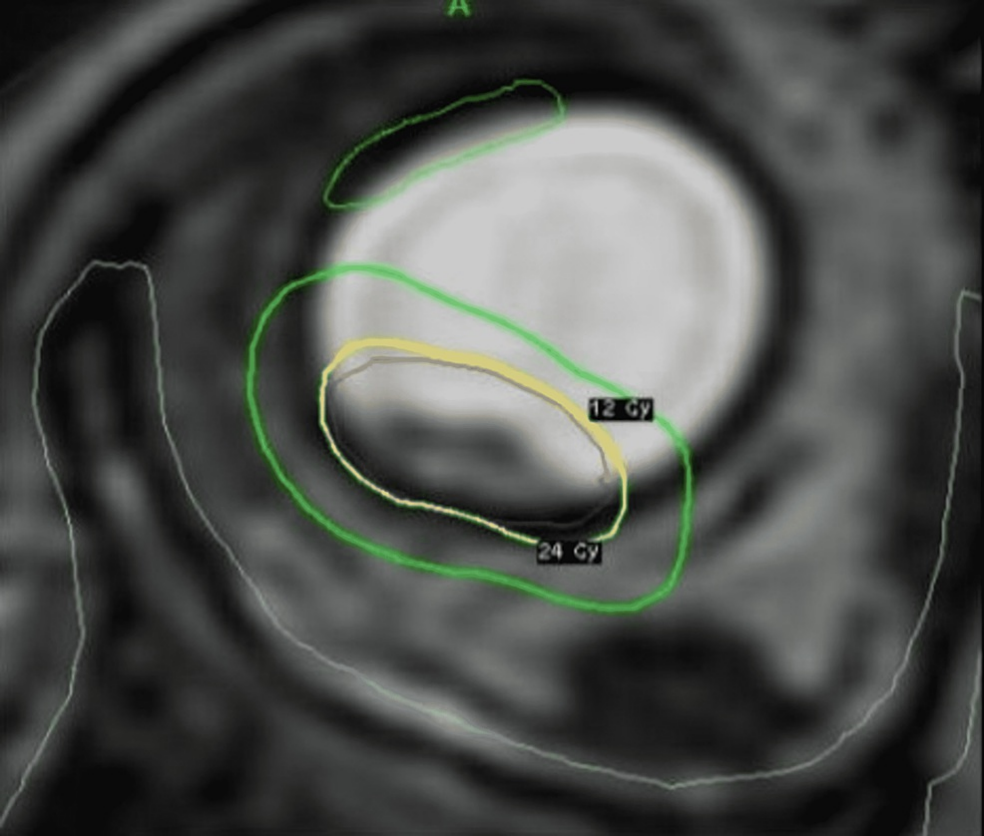
Treatment planning

**Figure 8:**
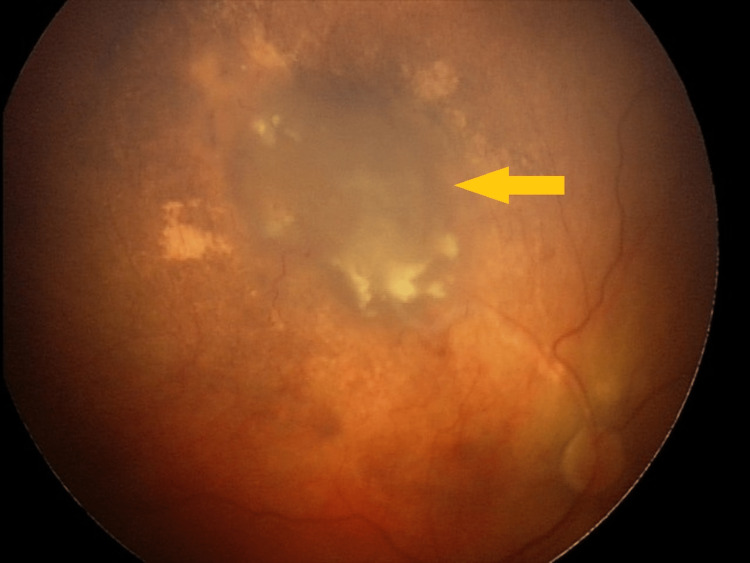
Incomplete tumor regression three months after GKRS GKRS - Gamma Knife® radiosurgery

**Figure 9:**
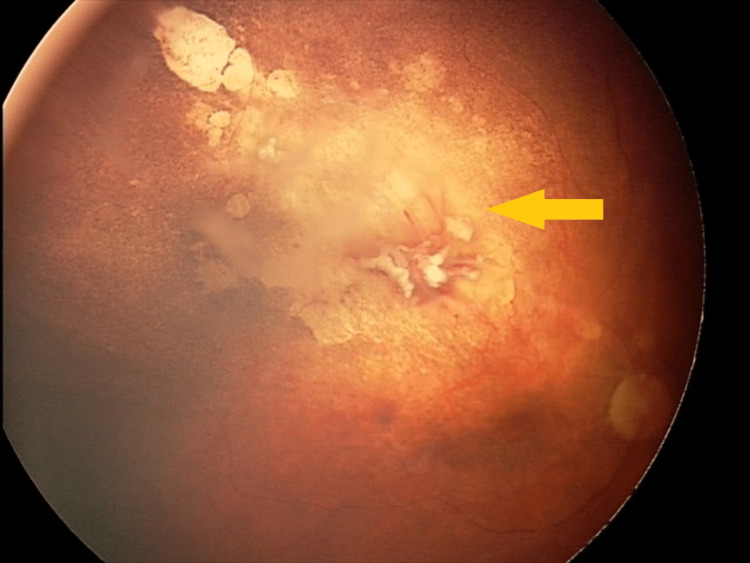
Complete tumor regression one year after GKRS GKRS - Gamma Knife® radiosurgery

Overall, 15 eyes (79%) were preserved, and four eyes (21%) were enucleated after GKRS with no signs of tumor recurrence and metastasis in the mean follow-up of 41 months.

No acute radiation side effects occurred in any patient after GKRS. Ten children (10 eyes, 53%) were diagnosed with vitreous hemorrhage from mild to severe. In four cases, vitreous hemorrhage resolved after conservative therapy, including catheterization of retrobulbar space. Six eyes developed total vitreous hemorrhage that obscured the eye fundus. Two eyes were enucleated as described above. Four patients' parents refused to remove the eye without confirmation of a viable tumor. In these children, pars-plana vitrectomy with melphalan irrigation was decided upon, and immediate enucleation with subsequent EBRT in the case of a viable tumor. Up to 15 months after GKRS, 25G vitrectomy with melphalan irrigation and lensectomy were performed, as reported earlier [20]. No active tumor was detected neither intraoperatively nor on histological examination. Within six to 38 months (mean of 19 months), all four eyes were stable with no intra- or extraocular relapse.

Three eyes presented with optic neuropathy one year after GKRS, and four eyes developed retinopathy. In all seven cases, patients were successfully treated with retrobulbar steroid injections. Radiation-induced cataract occurred in two eyes 16 months after GKRS; these patients underwent phacoaspiration with intraocular lens (IOL) implantation with no signs of tumor recurrence and metastasis in the mean follow-up of 17 months. There were no cases of secondary glaucoma or keratopathy in our study.

Successfully treated eyes were stable within 41 months (from seven to 74 months).

## Discussion

Over recent years, Rb treatment has shifted toward different types of chemotherapy that have assumed a major role in its management. At the same time, some limitations of chemotherapy exist. Ocular side effects and systemic toxicity are common and may stop subsequent treatment [21-23]. Moreover, Demirci et al. showed that in the majority of enucleated eyes previously treated with chemotherapy, viable retinoblastoma cells were detected within the tumor [24]. The main obstacle to the successful treatment of Rb was confirmed to be resistance to chemotherapeutic agents. All routes of chemotherapy delivery were held in all cases presented, and tumor chemoresistance was seen in 11 of them (Table 1). One patient showed incapacity of IAC due to ophthalmic artery spasm, which has been described by Shields et al. as occurring in 2% of cases; in one case, stroke occurred after IAC, which made it impossible to obtain complete tumor regression. IAC is a rather safe procedure nowadays; however, such systemic complications, even though they are not very frequent, were previously reported [25].

It is important to underline that GKRS in our study was used as rescue therapy and the last resort to save eye in selective cases. Despite that, GKRS proved to be a highly effective method of organ-preserving treatment (76%) in eyes with the inefficiency of multiple treatment modalities. Indications for GKRS in our study were Rb chemoresistance, local treatment failure and tumor recurrence, inability to perform IAC due to the previous ophthalmic artery spasm or stroke, Hagemann's disease, and tumor localization near the optic nerve head. In all of these cases, conventional ERBT could be used as an alternative treatment option, which was not considered due to its severe complications mentioned above.

EBRT has been successfully used for a long time in Rb management [3-6], especially as a salvage treatment alone or in combination with chemotherapy. Despite the great success of complete tumor control, and globe and vision preservation, EBRT has been found to have severe complications [7-11]. On the one hand, radiation affects the growth of soft tissue and orbital bones, leading to mid-facial hypoplasia of Rb survival; on the other hand, it has been proved to increase the life-long risk of secondary cancers in children with Rb1 mutations [7,8]. Therefore, EBRT was excluded in the presented cases, given the complications mentioned above. GKRS was proposed as an alternative to enucleation. Several publications have reported the successful use of proton beam radiotherapy, stereotactic radiotherapy, and intensity-modulated radiotherapy in children with Rb [12-19]. However, these methods are associated with the need for multiple (up to 30) sessions in children under general anesthesia, the difficulty of immobilizing the eye at each session, and the cost of treatment (concerning proton beam therapy). A review of medical databases revealed no information on single-fraction GKRS for Rb treatment.

The conventional EBRT therapeutic dose for Rb is 45-50 Gy within 20-22 fractions. There have been reports of successful treatment with doses lower than 36 Gy [5-7,16,19]. Due to the lack of experience with GKRS in Rb management, the issue of choosing optimal radiation doses was quite challenging, especially at the beginning of our study. Optimal doses had been selected empirically based on the experience of radiosurgery of other tumors, such as brain metastasis [26,27]. At the moment, we consider 20-22 Gy 50% margin isodose to be the optimal prescribed dose during GKRS.

The dosethreshold for bone growth inhibition is not precisely known, but it may be as low as 20 Gy with 1.8-2 Gy per session [15-19]. In our cases, the median dose to bones was 12.3 Gy (from 4.7 to 23.2 Gy) given in one-day treatment. The risk of possible second cancers and skull damage is extremely decreased compared to that of EBRT due to high conformity and selectivity of dose distribution in the case of GKRS. However, it is important to admit that correct dose comparison of single- and multiple-fraction radiation therapy is not possible, and this question needs further studies.

Based on the high conformity of GKRS plans, which are usually comparable to the characteristics of IMRT plans, we assessed the risk of secondary cancers in the irradiation zone as extremely low, taking into account other factors as well; firstly, radiation therapy was not carried out in children younger than one year - the most dangerous age in terms of the risk of radiation-induced tumors development [10], and secondly, only seven children had Rb1 gene mutation. The actual assessment of risks of second cancers development and orbital bone growth retardation is currently unavailable due to the short catamnesis period.

Results of our relatively small patients group showed that GKRS helps to achieve local tumor control, both for retinal and vitreous tumors. Although various kinds of radiation complications generally occurred in 79% of cases, their degree should be taken into account, as well as multiple treatment modalities that have their own toxicity to the vessels of the eye. The relationship between the frequency of these complications with doses to critical structures, pretreatment time, and other risk factors is subject to further analysis, which results will improve the planning of the technique and reduce the frequency of complications. It should be noted that in most cases, these complications are resolved after medical therapy. In addition, surgical techniques like pars-plana vitrectomy with melphalan irrigation and phacoaspiration of cataract can be an effective way to obtain ocular media transparency and adequate tumor control in selective cases.

Limitations of this study were the following: relatively small patient group and its' retrospective nature.

## Conclusions

GKRS proved to be a highly effective method that allows to achieve tumor control and to preserve eyes in 79% of cases. It is important to admit that the procedure was well-tolerated in all cases, with no acute radiation side effects in any patient. Despite that, various kinds of radiation complications generally occurred in 79% of cases; their degree should be taken into account, as well as multiple treatment modalities that have their own toxicity to the vessels of the eye. The relationship between the frequency of these complications with doses to critical structures, pretreatment time, and other risk factors is subject to further analysis, which results will improve the planning of the technique and reduce the frequency of complications. In conclusion, our experience showed that single fraction GKRS might be a reasonable salvage treatment for resistant and recurrent Rb as an alternative approach to enucleation in selected cases. Although the number of patients is small, a greater number of results and a longer follow-up time are expected to confirm this so emphatically that GKRS should be considered in retinoblastoma management.
